# Triple combination therapy and zeaxanthin for the treatment of neovascular age-related macular degeneration: an interventional comparative study and cost-effectiveness analysis

**DOI:** 10.1186/s40942-015-0019-2

**Published:** 2015-11-09

**Authors:** R. Joseph Olk, Enrique Peralta, Dennis L. Gierhart, Gary C. Brown, Melissa M. Brown

**Affiliations:** 1 The Center for Value-Based Medicine®, Box 335, Flourtown, PA 19031 USA; 2 The Retina Center of St. Louis County, St. Louis, MO USA; 3 ZeaVision, 680F Crown Industrial Court, Chesterfield, MO 63005 USA; 4 Retina Service, Wills Eye Hospital, Jefferson Medical College, philadelphia, PA USA; 5 Research Department, Wills Eye Hospital, Jefferson Medical College, philadelphia, PA USA

**Keywords:** Zeaxanthin, Macular pigment, Combination therapy, Neovascular age-related macular neovascularization, Cost-effective

## Abstract

**Background:**

Reports of triple combination therapy for neovascular age-related macular degeneration (AMD) suggest a benefit, as do reports for zeaxanthin. An interventional comparative study was thus undertaken to evaluate the efficacy of triple combination therapy with and without zeaxanthin, as well as the economic viability of the therapies.

**Methods:**

The cases of 543 consecutive eyes of 424 patients with subfoveal choroidal neovascularization (CNV) secondary to AMD were reviewed. All eyes were treated with triple combination therapy (triple therapy) consisting of: (1) reduced-fluence photodynamic therapy with verteporfin, (2) intravitreal bevacizumab and (3) intravitreal dexamethasone. Therapy was repeated as necessary. One cohort of patients was also given supplementation with 20 mg of oral zeaxanthin (Zx) daily.

**Results:**

The triple therapy group without Zx received a mean of 2.8 treatment cycles and 87 % of patients had stable or improved vision at 24 months. In the triple therapy group with Zx, the mean number of treatment cycles was 2.1, with 83 % of patients having stable or improved vision at 24 months. At 24 months, CNV developed in 12.5 % of fellow eyes treated with triple therapy alone; CNV developed in 6.25 % of eyes treated with triple therapy with Zx (p = 0.03). An average cost-utility analysis revealed that triple therapy was cost-effective with a cost-utility ratio of $26,574/QALY, while triple therapy with Zx was more cost-effective with an average cost-utility ratio of $19,962/QALY. The incremental cost-utility analysis assessing the addition of Zx to triple therapy disclosed Zx supplementation was very cost-effective at $5302/QALY. When it was assumed that triple therapy with Zx reduced fellow eye CNV development by 30.3 %, the incremental cost-utility dropped to (−$6332/QALY), indicating that adding Zx to triple therapy yielded greater patient value, and was also less expensive than using triple therapy alone.

**Conclusions:**

Triple therapy is comparatively effective and cost-effective. Considerably less treatment is needed than reported in monotherapy studies. The addition of oral Zx appears to further reduce the treatment cycles required, and possibly reduce the risk of CNV development in the fellow eye.

## Background

Age-related macular degeneration (AMD) is the leading cause of vision loss in adults over age 50 [1, 2]. The most severe vision loss occurs from choroidal neovascularization (CNV) destroying macular structure and function. Vascular endothelial growth factor A (VEGF-A) has been identified as promoting CNV [3–5]. Studies have also noted the inverse association between the xanthophyll carotenoid zeaxanthin (Zx) and AMD pathogenesis [6, 7].

Current treatment options for neovascular AMD are multiple, but each has deficiencies. Photodynamic therapy with verteporfin (PDT) directly targets CNV. While it has been shown to be effective at selective destruction of vascular endothelial growth cells, the treatment incites an injured cell response that is counter-productive [8, 9].

Many studies have established anti-VEGF efficacy for CNV and AMD. The ANCHOR [10] and MARINA [11] studies demonstrated the benefit of monthly injections of ranibizumab, the CATT [12] study showed the equivalency of bevacizumab and ranibizumab, and the PIER and PRONTO Studies [13] showed decreased frequency of treatment can still be effective. Overall cost and the burden of frequent injections, however, diminish satisfaction with this therapy.

The presence of an inflammatory component to AMD is now well established [14]. Thus, there is also a rationale for the use of corticosteroids for treating neovascular AMD [15, 16].

A limited number of studies have begun to evaluate the possible synergy created through a combination of the three treatment modalities: PDT, anti-VEGF therapy and corticosteroids [15, 16]. These studies seem to balance efficacy and treatment burden.

Major epidemiology studies [17–20] have shown that both higher dietary and serum levels of lutein and Zx are associated with significantly lower odds ratios of AMD. The AREDS Study Group [19] found higher dietary intakes of lutein and Zx reduced progression of both wet and dry AMD. The findings were so significant that the AREDS II was undertaken to study these specific effects.

In this study, we sought to further explore triple combination therapy (triple therapy) potential for CNV, with the thought of reducing treatment burden at a reduced cost compared to monotherapy treatments. In addition, we investigated the added effect of oral Zx. This paper is NOT a randomized clinical trial, but rather an interventional comparative study of two cohorts of patients treated for neovacular AMD. One cohort was treated with triple therapy alone, the other with triple therapy plus oral Zx. Recognizing the limitations of an interventional comparative study, the investigation was also undertaken to assess whether a prospective, randomized, controlled trial should be considered.

## Patients and methods

This comparative interventional study enrolled 424 participants with 543 eyes with neovascular AMD. The consecutive triple therapy without Zx patients were treated initially. When the lead author discovered the theoretical benefits of oral Zx, this was added to triple therapy. Thus, the triple therapy with Zx cohort participants were all enrolled after all of the cohort without Zx participants were already enrolled and had begun treatment.

*Inclusion criteria* Participants with classic, minimally classic, and/or occult subfoveal CNV were enrolled. Only eyes with macular blood, subretinal fluid, and/or retinal edema with characteristic CNV findings confirmed by fluorescein angiography, optical coherence tomography (OCT) or indocyanine green angiography were included.

*Exclusion criteria* Eyes with greater than 12 optic disc areas of CNV were excluded. Eyes with less than 20/400 vision were also excluded. The presence of blood was not an exclusion feature unless it covered greater than 12 disc areas.

*Demographic features* Two hundred seventy-nine participants were female and 145 were male. The mean age was 80 years, with a range from 50 to 99 years. All patients were taking a multi-vitamin (usually Centrum Silver; Pfizer) and an AREDS I antioxidant regimen (usually PreserVision; Bausch & Lomb) during the trial.

*Triple therapy patients without zeaxanthin* In this group there were 290 eyes of 210 patients; 182 were female and 58 male, with an age range of 50–99 and a mean age of 82. Eighty patients presented with bilateral CNV and 130 patients had unilateral CNV. Two hundred sixty-one eyes (90 %) were followed for 12 months and 206 eyes (71 %) for 24 months. Baseline visions ranged from 20/30 to 20/400, with a mean LogMAR visual acuity of 1.12 [20/250]. Patients received an intravitreal injection of 1.25 mg of bevacizumab at the initial visit, 1000 micrograms of intravitreal dexamethasone within 1 week, and reduced-fluence (PDT), usually within 2 weeks from baseline. PDT therapy utilized 25 mJ for 83 s. This protocol constituted one cycle of treatment. Reduced-fluence PDT was selected because full-fluence appears to cause more long-term retinal thinning [21]. Other authors have used half-fluence PDT with triple therapy with success [22].

After the initial treatment cycle, patients were re-examined within 4–6 weeks. Once deemed stable, follow-up was undertaken every 6–8 weeks in year one and every 8–12 weeks in year two. Retreatment was based on the presence of any of the following: subretinal fluid/blood on clinical exam, intraretinal or subretinal fluid on optical coherence tomography (OCT), decrease in vision, late leakage on fluorescein angiography, or an occult plaque on indocyanine green angiography. When retreatment was necessary, triple therapy was given.

*Triple therapy patients with Zx* The second consecutive series comprised 253 eyes of 214 patients, 127 female and 87 male. Their ages ranged from 53 to 97, with a mean of 80 years. One hundred twenty-one eyes (94.1 %) were followed for 12 months and 93 eyes (72.4 %) were followed for 24 months. Visual acuity ranged from 20/30 to 20/400 at baseline, with a mean initial LogMAR visual acuity of 1.00 (20/200). This second cohort was treated with triple therapy identical to the first. In addition, participants were given oral zeaxanthin, 20 mg, daily (Eye Promise Zeaxanthin, ZeaVision), for 2 years. Retreatment criteria were the same.

### The economic model

Incremental and average cost-utility analyses were performed using a Value-Based Medicine^®^ (standardized) cost-utility model [23, 24]. Patient time tradeoff utilities, a third party insurer cost-perspective and national, average, Medicare Fee Schedule costs (Table 1) were utilized. The base case employed an incremental cost-utility analysis comparing triple combination therapy with Zx to triple combination therapy without Zx for neovascular AMD. Average cost-utility analyses compared triple therapy with Zx to no treatment and triple therapy to no treatment.

**Table 1:** Average national medicare costs in 2015 US nominal dollars

Intervention	CPT code	Cost per treatment
Visudyne dye for PDT therapy	J3396	$1613
Intravitreal bevacizumab, 1.25 mg	J9035	$68
Intravitreal dexamethasone, 1 mg	J1100	$10
Photodynamic therapy physician fee	67,221	$298
Intravitreal injection of medication	67,028	$106
Fundus photography	92,250	$79
Intravenous fluorescein angiography	92,235	$111
Indocyanine green angiography	92,240	$256
Optical coherence tomography	92,134	$46
Ophthalmological services, medical examination and evaluation	92,004	$151
Ophthalmological services, medical examination and evaluation	92,012	$87
Ophthalmological services, medical examination and treatment	92,014	$126
Eye Promise Zeaxanthin^a^, Zea Vision, 20 mg per day, 1-year cost	NA	$360

*CPT* current procedural terminology, the interventional classification utilized by Medicare, *NA* not applicable

^a^Not included within the Medicare CPT codes

#### Model time frame

A nine-year time frame was used, the mean life expectancy for the average 81-year-old in the combined cohorts. A LOCF (last observation carried forward) methodology was employed from 25 months to 9 years. The base case assumed oral Zx was continued for 9 years.

A theoretical control cohort for both cohorts was created using data from Shah and DelPriore [25]. They analyzed control cohort visions from six randomized, neovascular AMD clinical trials with a Lineweaver-Burke model, demonstrating the mean vision associated with untreated neovascular AMD correlated with the time since onset of CNV. By the ninth year, the mean vision associated with untreated neovascular AMD deteriorated to 20/640^+1^ (Table 2).

**Table 2:** Mean visual acuity in the triple combination therapy with zeaxanthin, triple combination therapy without zeaxanthin and control cohorts

Year	Triple combination therapy with zeaxanthin cohort	Triple combination therapy cohort	External control cohort (Shah and DelPriore)
1	20/200	20/250	20/200
2	20/160 + 2	20/200 + 1	20/250 − 2
3	20/160 + 2	20/200 + 1	20/320 − 2
4	20/160 + 2	20/200 + 1	20/400 − 1
5	20/160 + 2	20/200 + 1	20/500 + 1
6	20/160 + 2	20/200 + 1	20/500 − 1
7	20/160 + 2	20/200 + 1	20/500 − 2
8	20/160 + 2	20/200 + 1	20/500 − 2
9	20/160 + 2	20/200 + 1	20/640 + 1

#### First-eye, second-eye models

The concept of first-eye, second-eye and combined-eye models was developed at the Center for Value-Based Medicine^®^ based upon primary patient data [23, 24, 26, 27]. They are based upon observations that vision-related quality-of-life most closely correlates with vision in the better-seeing eye. The first-eye model indicates the first eye is under treatment and the second eye is unaffected by the disease, neovascular AMD herein. The second-eye model indicates the first eye already has vision loss from the untreated disease. As clinicians know, losing vision in a second eye in addition to the first is devastating if untreated. Thus, the second-eye model yields the greatest patient value gain with therapy. The combined-eye model is simply the weighted average of the first-eye and second-eye models. The combined-eye model was used for the base case cost-utility analysis herein.

#### Patient preference-based comparative effectiveness

Study data were converted to a patient preference-based format to quantify quality-of-life using time tradeoff utilities obtained from over 1100 interviews with ophthalmic patients [23, 24]. Utilities are often referred to as patient preferences since patients can prefer to trade something of value (theoretical time of life) to hypothetically improve their health state, or prefer not to trade and keep the same health state. Excellent validity [28] and reliability [29] have been demonstrated for these utilities. They have been utilized in numerous peer-reviewed papers by the authors [26, 27, 30–34] and other researchers [35–37].

Vision utilities range from 1.00 (bilateral vision ≥20/20 permanently) to 0.00 (death). As vision in the better-seeing eye decreases, the associated utility and quality-of-life decrease. The lowest vision anchor utility is 0.26, which correlates with bilateral no light perception [30]. Our vision results were converted to utilities, then to QALYs (quality-adjusted life-years). People accrue QALYs as they live. The total QALY accrual is calculated by multiplying (utility) × (years lived at that utility). For example, living at a utility of 0.80 for 3 year accrues 2.40 QALYs (0.80 × 3), and so forth.

Using the average life expectancy of 9 years [38], we calculated how many QALYs were accrued by the mean triple therapy with Zx patient versus the mean triple therapy patient. Adverse events included the disutility associated with intravitreal injection, predominantly discomfort up to 24 h. The utility of 0.89 associated with this condition was obtained from 68 patients in the Center for Value-Based Medicine^®^ Time Tradeoff Utility Database, a compendium of over 50,000 patient utilities with acquisition approved by the Wills Eye Hospital Institutional Review Board. There were no cases of endophthalmitis. One vitreous hemorrhage requiring vitrectomy and one retinal detachment occurred, both in the triple therapy cohort. Nonetheless, the incidences of these adverse events were not significant (p = 0.50, Fisher Exact Test), and therefore not included in the base case analysis. A 0.002 QALY loss previously associated with PDT adverse event disutilities was used [31].

#### Cost-utility (cost-effectiveness) analysis

The model outcome was $/QALY, or dollars expended per QALY gained from the intervention. This is the cost-utility (cost-effectiveness) ratio (CUR). Per the Panel on Cost-Effectiveness in Health and Medicine, all QALY accruals and costs were discounted at 3 % annually [39].

#### Statistics

Statistics comparing ratios of patients with second eye progression to neovascular AMD were performed with the Chi square test, which was also used to evaluate other categorical variables. Linear variables were compared using the Student’s *t* test. (Microsoft Excel, Bellevue, Washington). Significance was presumed to occur at p < 0.05.

The SSM Health Care Institutional Review Board approved this study (approval number 14-07-0540. It adhered to the Helsinki Declaration of 1975, as revised in 1983. All participants signed an informed consent form. A four-year, prospective, randomized, clinical trial comparing triple therapy and triple therapy with Zx for treating neovascular AMD is currently in its third year. See Clinical Trials.gov (Identifier: NCT 01527435).

## Results

### Clinical features

**Fig. 1:**
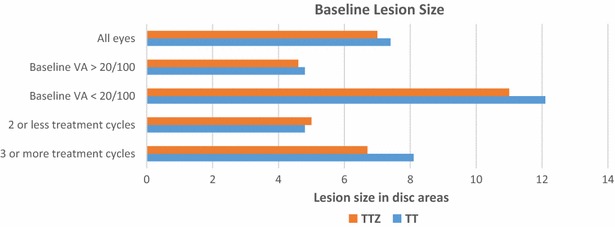
Baseline lesion size and treatment cycles. Baseline lesion size corresponded significantly to number of treatment cycles required and decreased visual acuity. *TTZ* triple combination therapy with zeaxanthin, *TT* triple combination therapy, *VA* vision. Baseline VA <20/100 = 20/200 or worse vision (p < 0.0001 for baseline VA >20/100 versus baseline VA <20/100, as well as for 2 or less treatment cycles versus 3 or more treatment cycles)

The mean baseline CNV size in the triple therapy cohort was 7.0 disc areas (DA) (SD = 3.5, 95 % CI 6.5–7.5) while that in the triple therapy with Zx cohort was 7.4 DA (SD = 3.5, 95 % CI 6.9–7.9) (p = 0.32) (Fig. 1). CNV size ranged from <1 DA to 12 DA. In both cohorts with unilateral neovascular AMD, over 90 % of fellow eyes had Age-Related Eye Disease Study, Category 3 AMD [40] with drusen >125 μm, typically with pigmentary changes.

The triple therapy cohort, mean visual acuity improved from baseline 20/250 to 20/200 at 12 and 24 months (Table 2), a mean gain of 6 EDTRS letters (p < 0.0001). Visual acuity was stable or improved in 79 % of eyes after 1 year and 87 % after 2 years. The mean number of treatment cycles was 2.1 over 1 year and 2.8 over 2 years. Eyes had a mean reduction in retinal thickness from 265 mµ (SD = 79, 95 % CI 252–278) to 244 mµ (SD = 66, 95 % CI 233–255), −21 mµ (−8.0 %) microns on OCT, at 2 years (p = 0.001) (Table 3).

**Table 3:** Central foveal thickness as seen on OCT

	Mean baseline CFT (μm)	Mean 2-year CFT (μm)	Mean change (μm)	% Thickness change (%)	p value
TT	265	244	−21	−8.0	p = 0.001
TTZ	287	232	−55	−19.2	p < 0.0001

*TT* triple combination therapy, *TTZ* triple combination therapy with zeaxanthin, *CFT* central foveolar thickness

Triple therapy plus Zx eyes had mean vision improve from baseline 20/200 to 20/160 at 12 and 24 months (Table 2), an average gain of 7 EDTRS letters (p < 0.0001). Eighty-three percent of eyes had stable or improved vision at 2 years, 27.5 % gained 15 letters or more on EDTRS, and 17 % were worse by 3 or more lines (p = 0.85 versus triple therapy cohort distribution). Overall, the mean number of treatment cycles was 1.6 at 1 year and 2.1 over 2 years. The addition of Zx reduced the mean treatment cycles by 24 % at 1 year and 32 % over 2 years. Eyes had a mean retinal thickness reduction from 287 mµ (SD = 70, 95 % CI 274–300) to 232 mµ (SD = 79, 95 % CI 223–241), a decrease of 55 microns (−19.2 %) on OCT at 24 months (p < 0.0001) (Table 3). In both cohorts, larger baseline lesion size correlated with increased treatment cycles (p < 0.0001) and decreased vision (p < 0.0001). Visual and anatomic results were compared between eyes with classic CNV and occult CNV. No statistical difference between these groups was seen across all parameters analyzed.

**Fig. 2:**
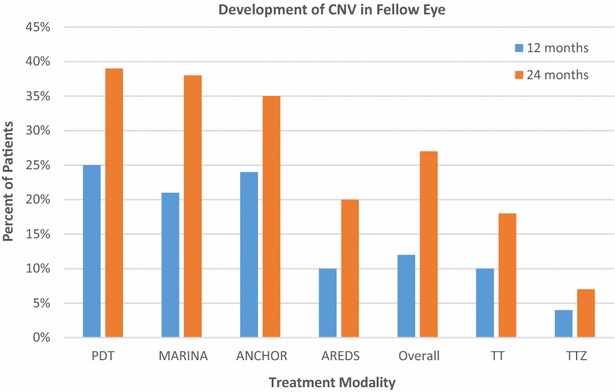
Choroidal neovascularization conversion in the fellow eye. The percentage of patients that converted to CNV in the fellow eye in the current study and other studies: Barbazetto et al. [41] for ANCHOR, MARINA and *PDT* photodynamic therapy with verteporfin, Overall numbers from a meta-analysis (Wong et al. Ophthalmology 2008;115:1524), *AREDS* Age-Related Eye Disease Study [19], *TT* triple combination therapy in current study, *TTZ* triple combination therapy with zeaxanthin in current study (*CNV* choroidal neovascularization). A 24-month comparison of conversion rates to CNV in the fellow eye in the TT and TTZ cohorts showed that zeaxanthin significantly decreased conversion in fellow eyes (p = 0.03). A 24-month MARINA and ANCHOR CNV conversion study in the fellow eye revealed a far greater 24-month rate in MARNA/ANCHOR than in our triple combination therapy cohort (p = 0.6 × 10^−6^) or the triple combination therapy cohort with zeaxanthin (p = 1.0 × 10^−14^)

The triple therapy cohort had 160 patients with CNV in the first eye and drusen in the fellow eye. Overall, 20 fellow eyes (12.5 %) developed CNV over 2 years, 12 in year one and 8 in year 2. In the triple therapy plus Zx cohort, 80 people had CNV in the first eye and drusen in the fellow eye. Overall, 5 fellow eyes (6.25 %) developed CNV over 2 years, 3 in year 1 and two in year 2 (p = 0.03 versus triple therapy cohort) (Fig. 2; Table 4).

**Table 4:** Choroidal neovascularization in the fellow eye developing from 0 to 24 months after baseline versus Barbazetto et al. [41]

	Conversions in current study (conversions/fellow eyes with dry AMD)	Conversions-Barbazetto et al. [41] (conversions/fellow eyes with dry AMD)	p-value
TT, 12 months	12/160 (20 %)	100/445 (22.5 %)	0.0001
TTZ, 12 months	3/80 (3.75 %)	100/445 (22.5 %)	0.00003
TT, 24 months	20/160 (12.5 %)	151/445 (33.9 %)	0.0000006
TTZ, 24 months	5/80 (6.25 %)	151/445 (33.9 %)	1 × 1.0^−14^

*TT* triple combination therapy, *TTZ* triple combination therapy with zeaxanthin, *CFT* central foveolar thickness

### Economic analysis

#### Patient value gain

QALYs accrued over 9 years by the triple therapy with Zx, triple therapy and control cohorts are shown in Table 5. The overall QALY gains for the cohorts are shown in Table 6. Utilizing the combined-eye model, triple combination therapy with Zx confers an 8.2 % quality-of-life (QOL) gain versus no therapy (p < 0.0001), while triple therapy confers a 6.0 % QOL gain versus no therapy (p < 0.0001) The highest QOL gain of 14.1 % occurs in the triple therapy with Zx cohort with the second-eye model.

**Table 5:** QALY (quality-adjusted life-year) accrual associated with the three cohorts over 9 years (discounted at 3 % annually)

Model	TTZ cohort	TT cohort	Control cohort [25]
First-eye model	7.202	7.050	6.749
Second-eye model	5.269	5.016	4.616
Combined-eye model	6.546	6.384	6.024

*TTZ* triple therapy with zeaxanthin, *TT* triple therapy

Combined-eye model = 66 % first-eye model and 34 % second-eye model, Zeaxanthin = Zx

**Table 6:** QALY and (percent patient value gains) for triple combination therapy cohorts associated with zeaxanthin use and with no zeaxanthin use

Model	TTZ vs. control cohort	TT vs. control cohort	TTZ vs. TT
First-eye model	0.453 (6.7 %)	0.339 (5.0 %)	0.115 (1.6 %)
Second-eye model	0.653 (14.1 %)	0.400 (8.7 %)	0.253 (5.0 %)
Combined-eye model	0.521 (8.2 %)	0.359 (6.0 %)	0.162 (2.5 %)
Combined-eye model	p < 0.0001	p < 0.0001	p < 0.0001

*TTZ* triple therapy with zeaxanthin, *TT* triple therapy

Combined-eye model = 66 % first-eye model and 34 % second-eye model, Zeaxanthin = Zx

A comparison of the two treatment cohorts reveals adding Zx enhances QOL by 2.5 % over triple therapy alone (p < 0.0001) (Tables 5, 6). This is the incremental patient value gain. Sixty-six percent of patients in the current study presented with a first-eye model and 34 % a second-eye model when calculating the combined-eye patient value gain. Nonetheless, each treated second-eye patient in the triple therapy with Zx cohort contributed 2.21× the value contributed by a treated first-eye patient. The less favorable, first-eye, vision utility data were taken from the Center for Value-Based Medicine Utility Database [23, 24, 26–34].

#### Costs

The costs associated with therapeutic strategies are shown in Table 7. The total discounted Zx cost per patient over 9 years was $2887. The total triple combination therapy with Zx cost per patient for the combined-eye model was $10,440. It dropped to $8222 when Zx was given for only 2 years instead of nine. The cost for triple therapy was $9540, intermediate between Zx given for 2 years and 9 years. The base case cost of $10,440 in the triple therapy with Zx cohort was distributed as follows: physician: $2521 (24 %), diagnostic testing: $815 (8 %), drugs other than Zx: $4176 (40 %), and Zx: $2887 (28 %).

**Table 7:** Nine-year costs for zeaxanthin and no zeaxanthin triple therapy (2015 US Real dollars, discounted at 3 % annually)

Model	No zeaxanthin treatment cohort	Triple combination therapy with zeaxanthin (9 years of zeaxanthin)	Triple combination therapy with zeaxanthin (2 years of zeaxanthin)
First-eye, second-eye or combined-eye model: the same for all	$9540	$10,440	$8222

Combined-eye model = 66 % 1st-eye model and 34 % 2nd-eye model

*TTZ* triple combination with zeaxanthin, *TT* triple combination therapy

#### Cost-utility (cost-effectiveness)

The base case, combined-eye, incremental CUR for the use of triple therapy with Zx referent to triple therapy alone was $5302/QALY (Table 8). This assumed Zx was used for 9 years. The average CUR for the use of triple therapy with Zx was $19,962/QALY, while the average cost-CUR for the use of triple therapy was $26,574/QALY.

**Table 8:** Cost–utility ratios of triple therapy and triple therapy with zeaxanthin

Model	TTZ vs TT	TTZ vs. control cohort	TT vs. control cohort
	Incremental CUR	Average CUR	Average CUR
First-eye model	$7470/QALY	$22,958/QALY	$28,142/QALY
Second-eye model	$3395/QALY	$15,926/QALY	$23,850/QALY
Combined-eye model	$5302/QALY	$19,962/QALY	$26.574/QALY

#### Sensitivity analysis (Table 9)

Sensitivity analysis assesses the variables of least confidence. In this instance, it demonstrated the incremental CUR for the triple therapy with and without Zx to range from (−$8148/QALY) when Zx was used for only the first 2 years to $23,892/QALY when Zx was used for 9 years, but had no benefit after 2 years. The cost of Zx must rise to $318/month for an incremental CUR of $100,000/QALY. The cost of Zx must rise to $513/month for a CUR of $158,000/QALY, or 3× the US Gross Domestic Product per capita.

**Table 9:** Sensitivity analysis

Model	Incremental cost	Incremental QALY gain	$/QALY
Therapeutic changes in the TTZ cohort			
Base case			
Zx daily for 9 years in the triple therapy with Zx cohort	$859	0.162	$5302
Zx daily for 2 years only in the triple therapy with Zx cohort	(−$1318)	0.162	(−$8148)
Two additional treatments: bevacizumab, PDT and dexamethasone, years 3–9 in both cohorts	$859	0.162	$5,312
Costs in both cohorts are doubled	$1718	0.162	$10,623
Zx cost over 9 years is doubled from $30/month to $60/month	$3746	0.162	$23,154
Zx cost/month for cost-utility ratio of $50,000/QALY	$150	0.162	0 $50,000
Zx cost/month for cost-utility ratio of $100,000/QALY	$318	0.162	$100,000
Zx cost/month for cost-utility ratio of $158,000/QALY(WHO upper limit for cost-effectiveness = 3× GDP per capita)	$513	0.162	$158,000
Altering patient value gains associated with TTZ			
Doubling patient value gains in both cohorts	$859	0.324	$2,656
Halving the patient value gain from Zx	$859	0.081	$10,623
Loss of Zx benefit after year 2 with 2 years of Zx therapy in the TTZ cohort	(−$1318)	0.036	(−$36,665)
Loss of Zx benefit after year 2, with 9 years of Zx therapy in the TTZ cohort	$859	0.036	$23,892
Zx incremental QALY gain for $50,000/QALY	$859	0.017	$50,000
Zx incremental QALY gain for $100,000/QALY	$859	0.0086	$100,000
Zx incremental QALY gain required for $158,000/QALY (WHO upper limit for cost-effectiveness = 3× 2015 US GDP per capita)	$859	0.0054	$158,000
Integrating the therapeutic QALY gain and costs saved by TTZ decreasing the onset of neovascular AMD in the fellow eye: combined-eye model			
Incremental cost-utility			
Zx daily for 9 years in the TTZ cohort vs. TT cohort—assumes that TTZ yields an absolute risk reduction of CNV in the 2nd eye of 30.3 %	(−$2291)	0.362	(−$6332)
Average cost-utility			
Zx daily for 9 years in the TTZ cohort vs. TT cohort: assumes TTZ reduces the absolute risk reduction of CNV in the 2nd eye by 30.3 %	7249	0.7212	$10,052

A negative cost-utility ratio indicates that neovascular age-related macular degeneration triple therapy with zeaxanthin provides greater patient value than triple therapy without zeaxanthin and is also less expensive than triple therapy without zeaxanthin.) (Dollars are 2015 US real dollars discounted at 3 % annually. QALYs are discounted at 3 % annually.

Incremental cost–utility of TTZ vs. TT cohorts, combined-eye model

*QALY* quality-adjusted life-year, *$/QALY* dollars expended per QALY gained, *PDT* photodynamic therapy with verteporfin, *GDP* gross domestic product, *TTZ* triple combination therapy with zeaxanthin, *TT* triple combination therapy, *Zx* zeaxanthin, *WHO* World Health Organization

Comparing our triple therapy with Zx cohort data to the higher, fellow eye, CNV conversion rates noted by Barbazetto et al. [41] there is a 2-year absolute risk reduction of 30.3 % (the 36.6 % incidence of fellow eye CNV in ANCHOR/MARINA minus the 6.3 % incidence of CNV with our triple therapy with Zx cohort) (p < 1.0 × 10^−14^). The triple therapy cohort also had less CNV conversions at 2 years (p = 0.6 × 10^−6^). The incremental CUR for Zx addition drops to (−$5601/QALY) with Barbazetto et al. data [41]. The minus CUR indicates that Zx with triple therapy dominates triple therapy by conferring more patient value for less cost. The average CUR for triple therapy with Zx drops to $10,052/QALY.

## Discussion

Our case series showed that triple therapy with Zx yielded a visual result superior to that of triple therapy alone. At the same time, it significantly decreased the onset of CNV in the fellow eye. That said, both triple therapy with and without Zx are superior to observation.

The series applied a combination of agents to treat subfoveal CNV in AMD. Anti-VEGF treatment is the mainstay in treatment for neovascular AMD. The MARINA study [11] was associated with monthly injections of ranibizumab. Visual acuity, ophthalmoscopic signs and diagnostic studies all showed significant benefits over 2 years of follow-up in patients with minimally classic or occult lesions. The ANCHOR study [10] found ranibizumab therapy more effective than PDT for predominantly classic, subfoveal CNV. The CATT Study noted that ranibizumab and bevacizumab similarly improved visual acuity [12]. It is difficult to compare patient value gain with those studies since they enrolled patients with a mean vision of 20/80, versus 20/200-20/250 in our study. There is a greater potential for value gain when CNV patients are treated earlier in their course with better vision [42].

Ongoing VEGF-inhibitor treatment, however, creates patient and healthcare system burdens. The patient burden may actually cause some to stop treatment before remission [43]. The incidence of serious ocular and systemic adverse events with anti-VEGF injections is low, but rates can increase when therapy is repeatedly applied [44]. In addition, VEGF may play a role in the survival and maintenance of RPE integrity [45].

PDT leads to selective cytotoxicity of vascular endothelial cells by producing oxidative radicals [46]. But despite an early angiographic effect suggesting disappearance of CNV, patients experience a mean visual loss of two ETDRS lines of vision during the first 6 months after treatment [8, 47]. Studies of the angiogenic effect of PDT show increased VEGF, VEGF-R and PEDF in eyes [9]. This suggests combination therapy with an anti-VEGF agent is reasonable.

Corticosteroids stabilize the blood retinal barrier and down regulation of inflammation [48]. In addition, they have anti-fibrotic and anti-angiogenic activity, the latter which can last for 3 months [48].

Multiple studies have suggested combination therapy addresses the mechanisms of disease progression. Combination PDT and ranibizumab therapy in AMD patients with CNV has been associated with CNV occlusion, reduced retinal edema and improved vision [49]. Wan and colleagues [50] treated 174 eyes with PDT, then intravitreal bevacizumab 30 days later. Over a mean 10 months, patients received an average of 3.0 bevacizumab injections and 1.4 PDT treatments. After stabilization, the mean treatment-free interval was 193 days, and 52 % of patients required no post-induction retreatment. Visual acuity improved at 2, 4, and 6 months.

In a number of population-based studies, lutein and Zx levels have been inversely associated with the risk of AMD. The POLA study [51] found high total plasma lutein and Zx reduced the risk of AMD by 79 %, with a particularly strong association between Zx and AMD. Subjects with high levels of plasma Zx had a 93 % reduced risk of AMD vs. those with low levels. Similar studies in China [52] and the UK [6], also showed Zx had a greater positive influence. In the UK study, a 50 % reduced risk of AMD was noted in people with high plasma Zx.

The reasons for Zx benefit are elusive. Evidence suggests a role for Zx as a filter to blue light, preventing it from entering the outer retina [53]. Short wavelength blue light contains energy, and filtering this light may limit metabolic insult. Zeaxanthin also serves as an antioxidant; it consumes singlet oxygen and may quench free radicals generated by normal retinal metabolism. Increased levels of Zx also inhibit VEGF levels [53, 54].

We recognize the limitations of consecutive case series versus a randomized controlled trial. However, our information suggests triple therapy with bevacizumab, dexamethasone and PDT is a viable option for wet AMD, and that oral Zx enhances that benefit. The percentage of stable or improved eyes in both of our cohorts (more than 80 %) is very encouraging. Oral Zx may also provide a protective benefit for the fellow eye. This requires further study, but suggests protection against CNV development could considerably preserve vision.

While some clinicians believe monthly injections of anti-VEGF drugs are not necessary with monotherapy, studies still show less visual acuity gains with less frequent dosing [12, 55]. Subjects receiving triple therapy plus Zx, however, kept the same vision from 12 to 24 months.

### Economic analysis

#### Cost-effectiveness

The use of oral Zx with triple combination therapy is very cost-effective, with an incremental cost-utility ratio (CUR) of $5302/QALY. No formal US formal cost-effectiveness criteria exist, but a CUR <$100,000/QALY is often thought cost-effective [24]. World Health Organization [56] criteria state a CUR <1x Gross Domestic Product (GDP) per capita (~US $53,000 [57]) is very cost-effective, while a CUR <3× GDP per capita (~US $158,000) is cost-effective. The National Institute for Health and Care Excellence (NICE) in the UK suggests a CUR <£20,000/QALY (~US $29,750) is cost-effective, although £30,000/QALY (~US $44,326) is sometimes allowed [58]. By any of the above criteria, triple combination therapy for neovascular AMD appears to be very cost-effective. The addition of oral Zx is more cost-effective yet. GDP per capita data suggest adding Zx to triple therapy would be cost-effective in 127 of the world’s 198 countries, even if healthcare costs were similar to the US [57].

## Conclusions

In summary, neovascular AMD triple therapy is comparatively effective and cost-effective. It requires considerably less treatment than needed with monotherapy. Adding oral Zx appears to further reduce the treatment cycles required, and possibly reduce fellow eye CNV development. Nonetheless, the results of a randomized trial will be critically important.

## Abbreviations

AMD: age-related macular degeneration
NVAMD: neovascular age-related macular degeneration
AREDS: Age Related Eye Diseases Study
QALY: quality-adjusted life-year
$QALY: dollars expended per quality-adjusted life-year gained
VBM: value-based medicine
CUR: cost-utility ratio
Zx: zeaxanthin
TT: triple combination therapy
TTZ: triple combination therapy with zeaxanthin
GDP: gross domestic product
CNV: choroidal neovascularization
VEGF: vascular endothelial growth factor
ETDRS: Early Treatment Diabetic Retinopathy Study
